# Strategic priorities and challenges in research software funding: Results from an international survey

**DOI:** 10.12688/f1000research.155879.2

**Published:** 2025-01-06

**Authors:** Eric A. Jensen, Daniel S. Katz

**Affiliations:** 1 National Center for Supercomputing Applications (NCSA), University of Illinois Urbana-Champaign, Urbana, IL, USA

**Keywords:** Research software, research policy, research funding, science systems, FAIR, open science

## Abstract

**Background:**

Research software is increasingly recognized as critical infrastructure in contemporary science. It spans a broad spectrum, including source code files, algorithms, scripts, computational workflows, and executables, all created for or during research. While research funders have developed programs, initiatives, and policies to bolster research software’s role, there has been no empirical study of how these funders prioritize support for research software. Understanding their priorities is essential to clarify where current support is concentrated and to identify strategic gaps.

**Methods:**

We conducted an online mixed methods survey of international research funders (n=36) to explore their priorities in supporting research software. The survey gathered data on the specific outcomes funders emphasize in their programs and initiatives for research software.

**Results:**

The survey revealed that funders place strong emphasis on developing skills, promoting software sustainability, embedding open science practices, building community and collaboration, advancing research software funding mechanisms, increasing software visibility and use, fostering innovation, and ensuring security.

**Conclusions:**

The findings highlight opportunities to enhance research software’s role through increased funder attention on professional recognition for software contributions and the non-technical, social aspects of research software sustainability. Addressing these areas could lead to more effective support and development of research software, ultimately benefitting the entire research ecosystem.

## Introduction

Research software is increasingly recognized as critical infrastructure in the contemporary research ecosystem ( Barker et al., 2022; Jensen & Katz, 2023b; Knowles et al., 2021; Hocquet et al., 2024). Research software spans a broad spectrum, including source code files, algorithms, scripts, computational workflows, and executables, all created for or during research ( Gruenpeter et al., 2021). With the growing reliance on computational methods across the sciences, supporting software development, maintenance, and impact is more essential than ever (e.g., Barker et al., 2020; Jay et al., 2021). For this reason, some research funders have developed programs, initiatives and policies to bolster research software’s role and impact ( Hertweck et al., 2024; Strasser et al., 2022). The research software landscape includes government and philanthropic funding sources ( Barker & Katz, 2022) that are seeking impact within the scientific ecosystem and beyond from their investments (e.g., Jensen et al., 2022). Research software is often open source and is sometimes tied to the larger open science movement that aims to improve transparency and reproducibility in research.

To date, there has been no empirical study of how software is supported by research funders, the aims of such support and the priorities that underpin it. This information is needed to clarify where current funder support is concentrated and highlight significant gaps. This study investigates the range of efforts by research funders to support the sustainability and impact of research software. The aim is for these findings to inform improvements in funder strategies that would bolster research software’s role and value for academic research and the wider society.

This paper draws on part of the dataset from this survey to address the following research question: What are the top priorities of international research funding organizations related to research software?

## Methods

This research was carried out using a survey combining qualitative and quantitative items. The survey was designed to investigate how research software funders support research software’s sustainability and impact.

### Survey design

The survey designed for this study began by collecting profile information, including institutional affiliation and job title. The survey gathered information about respondents’ organization’s initiatives, policies, or programs to support research software. The range of questions yielded too much data for one article. In this article, we focus exclusively on the results generated via an open-ended question asking about the top priorities for the respondents’ organizations’ support for research software: “What are your organization’s top priorities related to research software?” (see Jensen, 2024 for the extended data underpinning this article). Four open-response text boxes were provided for respondents to indicate and list these priorities.

Clearly, potentially relevant variables were not included in the survey design for this exploratory study. These include details on the exact amount of funding spent on research software and other details about which many research funders would be unable to readily report either because the information is not actively tracked or relevant data do not currently exist.

### Sampling

This survey was aimed at international research funders, including governmental and non-governmental (e.g., philanthropic) funders. A list of contacts to invite to participate in this survey was created based on participation in the Research Software Association (ReSA) and responsibility for research software funding known to the authors. ReSA is an international organization involving research policymakers, funders and organizations that recognize software’s importance in their work. This means that a ReSA-focused sample is likely to over-represent research funders who already view research software as important. For this reason, this paper does not claim that its findings generalize to the global population of research funders; rather, the claims focus on current patterns in research software funding. This initial list of people was refined, with removals based on individuals having moved to unrelated professional roles or being unavailable long-term, for example, due to personal issues.

The final, refined contact list comprised 71 people. After removing individuals when a member of their organization already provided a complete answer or when the person turned out to no longer be working on a relevant topic or to be otherwise unavailable (total of n=30), 41 people remained. Five of these individuals did not complete the survey, while 36 people (representing 30 research funding organizations) did, yielding a response rate of 88%. Fully completed survey responses were not required for individuals to be retained in the sample, resulting in varied sample bases across survey questions.

The sample includes research funders in North and South America, Europe, Oceania and Asia, but over-represents North America and European funder representatives. Some participating funders cover a broad spectrum of disciplines, while others focus on a particular domain such as social science, health, environment, physical sciences or humanities.

**Table:** 

Continent	Count
North America	15
South America	4
Europe	12
Oceania	3
Asia	1

The respondents represented research funders supported by governmental (n=26), philanthropic (n=6) and corporate (n=1) resources.

Respondents’ job titles span the following categories: *Senior Leadership and Executive*, such as a Vice President of Strategy; *Program and Project Management*, such as Senior Program Manager; *Planning and Business Development*; *Scientific, Technical and IT*, such as Scientific Information Lead.

Most respondents 72.7% (n=24) answered ‘Yes’ to the question, “Has your organization established any policies, initiatives or programs aimed at supporting research software?”, while 18.2% (n=6) said ‘No’ and 9.1% (n=3) ‘Unsure’.

### Data collection, management and analysis

Data collection took place from December 2023 to May 2024. The mean completion time for the detailed survey was 28 minutes and 13 seconds.

The data were cleaned and prepared for analysis by removing any identifiable respondent details. The data analysis process followed a standard thematic qualitative analysis approach (e.g., Jensen & Laurie, 2016). This involved first identifying themes and organizing the data accordingly. Dimensions of each theme were identified where relevant. Then data extracts were selected from the survey responses associated with each theme and theme dimension. In line with qualitative research methodology, the focus is on presence or absence of ideas in the survey responses, not quantification. However, prevalence has been used to organize the results section, as the more prevalent themes also come with a more extensive set of data extracts and potential dimensions. The results section below presents each theme in turn, with data extracts providing evidence to support the description of the themes.

## Results

The survey reveals several priorities driving research funders’ support for research software. These themes are presented below, ordered from the most to least prevalent categories. Many of these categories overlap, so the boundaries between themes are porous. After each point is explained below, a verbatim data extract in the form of a block quotation from the survey responses is presented to substantiate the explanation.

### Skills

The first identified theme, *Skills*, shows funders are prioritizing equipping researchers and other research professionals with competencies in software development, maintenance, and sustainability best practices.

Supporting the training and retention of qualified staff to ensure human expertise and support within and between institutions.

Funders were keen to ensure sufficient skilled personnel capable of writing and maintaining software.

Skills - ensuring that there are enough skilled people writing software.

Such skill development was viewed as foundational for research software as a field.

Building the necessary skills, training, infrastructure, and incentives to support the growth of the [research software] field.

Respondents noted a priority focus on researchers’ software skills, seeking to support “train [ing] researchers to cope with research software on different levels.”

Training: We have an active initiative to train researchers to build software and digital research infrastructure.

However, international funder priorities also included software-related skills for other research professionals.

Invest in the training of both professional research and support staff to be able to reuse, develop, and maintain sustainable research software.

Overall, the funder representatives indicated that training and skills development support was an important pathway to advancing research software.

### Software sustainability

*Software Sustainability* emerged as a significant priority for research software funders. Sustainability means “the software will continue to be available in the future, on new platforms, meeting new needs” ( Katz, 2024). Indeed, the term *sustainability* was frequently used to answer this question about top priorities for research software funding, such as “supporting research software sustainability” or “identifying pathways to sustaining the research software.” In this section, we focus on non-financial aspects of software sustainability that respondents highlighted. Indeed, this priority was expressed in many ways.

Sustainability and reuse of software. How can we reduce legacy debt and ensure that research software is continuously improved and reused.

Reducing waste by maximizing the use of existing software was a notable focus evident in funders’ responses.

Fund research software sustainability, and in particular incentivize the reuse and improvement of existing research software by providing funding to improve existing software.

Some responses evinced the view that sustainability needed to be supported because it was part of ‘best practice’ in research software: “Promote the best practices for the production of sustainable research software.” Others focused on keeping software relevant and useful as a pathway to sustainability: “Support improvements to essential open source tools in [a specific field of] research”. Another response highlighted the importance of having standardized measures and practices to ensure sustainability.

Establish metrics and best practices for software sustainability and integrate these into [the] software development lifecycle.

In sum, the responses signaled funders’ full-throated commitment to ensuring research software’s long-term viability and continuous improvement (also see Barker et al., 2022).

### Open science

The theme of *Open Science* highlights respondents’ emphasis on promoting open sharing, reuse, and accessibility of research software, adhering to the principles of open science. This theme is evident in the responses of research software funder representatives, who highlighted the importance of “open source practices” in fostering a collaborative and transparent research environment.

One respondent articulates the goal of promoting open sharing and reuse through significant initiatives, stating, “To promote the open sharing and reuse of research software, mainly through the European Open Science Cloud.” Many funder representatives highlighted maintaining, improving, and “making research software reusable” in their survey responses. This indicates a strategic effort to leverage established platforms to facilitate research software’s open distribution and reuse, thereby enhancing its accessibility and impact.

One respondent took an expansive view of what was included in efforts to integrate open science principles within research software: “Open Science including Accessibility, Inclusion, Reproducibility, Recognition, and security.” Another respondent called for a more explicit integration of research software into open science policies, saying, “Include research software more explicitly in our open science policies.” This points to the need for clear policy frameworks that explicitly address the role of research software in open science, ensuring that it is systematically included and supported (e.g., see Jiménez et al., 2017). This set of responses reflects a commitment to fostering an environment where a wide range of users and developers can contribute to and benefit from open-source research software.

### Building community and collaboration

The theme of *building community and collaboration* among research software funders encompassed three main dimensions: Enhancing community engagement, fostering international collaboration, and sharing best practices through professional networks. These community-related priorities were ultimately aimed at spreading and embedding good practices in research software.

The emphasis on community engagement highlights the importance of involving diverse groups in developing and maintaining scientific software. Funders prioritize approaches that ensure high-quality software through community involvement. They seek to promote opportunities that engage new communities in software development, making these resources accessible to under-represented communities and broadening participation in data science.

Promote opportunities to engage new communities in software development and make these resources accessible to under-represented communities interested in data science.

Another respondent emphasizes the importance of inclusivity in open source communities of practice:

Supporting diverse participants (users and developers) in scientific open source (research) software.

Additionally, there is a focus on community-focused software development and dissemination, ensuring that the processes and outcomes of software projects align with the community’s needs and contributions.

Enhance community-focused software development and dissemination.

International collaboration was identified as another critical dimension, focusing on creating a global network for software production. Funders aimed to facilitate work across institutions to prevent duplication of efforts and ensure that lesser-resourced institutions can equally benefit from open-source software, whether it be code, databases, or extensive infrastructure. This collaborative approach leveraged collective expertise and resources, promoting efficiency and innovation.

Enabling work across institutions so that there is no duplication of effort and lesser-resourced institutions can benefit from OSS (whether bits of code, databases, or huge infrastructure) as much as the larger institutions.

Moreover, fostering international collaboration is seen as a way to enhance the production of research software by tapping into a diverse pool of global expertise.

Foster international collaboration in the production of research software.

Funders prioritized disseminating best practices as a means of building robust communities around software development. Respondents emphasized the need for networks to share successful strategies and methodologies, ensuring that effective practices are widely adopted. This was aimed at contributing to improving research software quality and community development.

Best practice and community - ensuring that there are networks to disseminate best practice and build communities.

In addition to promoting best practices, there is a focus on developing community-based approaches for ensuring and improving the quality of scientific software.

Development of community-based approaches for ensuring and improving the quality of scientific software and code.

This response shows that funders linked community-building goals with practical concerns about continuous improvement and quality assurance of research software.

### Advancing research software funding

The theme of *advancing research software funding* encompasses “funding new research software”, strategic planning of funding support, and fostering collaborative efforts to improve software impact.

Funders noted the need for financial resources to support research software, for example, by “creating instruments to fund the production of high-quality research software.” They also emphasized the importance of developing plans to guide funding decisions over the medium term.

Our primary priority is developing a strategy for how to fund [research software for the] next [several years].

Such a forward-looking approach is undoubtedly challenging in the constantly evolving research software landscape.

Collaboration also emerged as a funder priority, with a systemic focus.

Fund joint developments of research software useful for the [research] ecosystem in collaboration with other institutions.

Overall, the theme of advancing research software funding highlighted funders’ commitment to creating strategic funding for research software.

### Increasing software visibility and use

Funders highlighted the need to make existing research software more visible to a broader range of researchers.

Provide visibility to successful computational projects from users of our platform, not only about the science but also about the software being developed.

Additionally, there was a focus on enabling existing software to facilitate wider adoption and make it more accessible and usable.

Make existing research software more *visible* [and] shape some of that software for wider adoption. [emphasis added]

One funder noted the need for a centralized research software portal where researchers could access information on supported platforms, tools, and services.

Developing a comprehensive catalog of currently supported [research software] platforms, tools and services that researchers can access via [a] single portal, including the cloud resources needed to run those services.

Another funder’s related suggestion also focused on bolstering the visibility of research software through a shared portal or database.

Develop a profile of funding and support programs for [research software], with a focus on those infrastructures that have a national and international impact, but also including emerging RS platforms that have not yet established their role/impact.

The aim of increasing the visibility of research software in this way was “encouraging the [scientific] field to use research software.” Ultimately, these efforts aim to enhance the overall impact of research software.

### Innovation

A small number of funders noted prioritizing integrating advanced technologies in software development. One funder noted the importance of “transitioning to accelerated compute” and maintaining pace with advancements in large-scale computing resources.

Cutting edge - ensuring that software is developed to keep pace with the next generation of large-scale compute and supports the best research.

A specific ‘innovation’ example mentioned was the “use of Generative AI in software engineering.” Such priorities reflected a forward-looking approach from a small subset of funders aiming to ensure that research software continues to support research effectively.

### Security

Funders raised the theme of “security” in the development and use of research software, including ensuring “data security,” enhancing software security, and conducting “Security Awareness Training.”

Security: Research software will not be able to be reused if IT departments are not confident that it is secure[,] even for Open science projects.

While infrequently mentioned compared to other themes and with limited detail in the responses, *security* was seen as a fundamental issue to be addressed for a small number of research software funders.

## Discussion

In this paper, we have described the priorities guiding research software funders. Here, we consider the implications of these priorities for advancing research software’s role in the scientific ecosystem.

Within the *skills* development priorities highlighted by respondents, several technical training topics to support the design, development and deployment of research software were mentioned. This funder priority aligns with an identified need for training in research software skills (e.g., Carver et al., 2022; Cosden et al., 2022). Moreover, this skills theme suggests that there is recognition among funders that there is a need to invest in the people who underpin research software (e.g., Hartley & Barker, 2023; Katz, 2021). However, the more social aspects of research software sustainability and impact were not explicitly mentioned by any respondents. Addressing such social aspects and non-technical skills is an essential need in the field. For example, research software projects need to be able to identify relevant parties to engage with, maintain effective communication with current and potential users, establish a robust framework for monitoring and addressing evolving needs in a user community, and ensure good user experiences with the software. A recently published report highlighted “UX design, product management, and community management” as examples of key roles that often go unfunded in open source research software projects ( Iacovou, 2024). Iacovou (2024) noted that this averred gap in available support was part of a broader need to address the critical social and community aspects of research software sustainability. While respondents in our survey explicitly noted community building and management, supporting the development of the skills underpinning this kind of work was not highlighted as a priority for research software funders.

*Software sustainability* emerged as a prominent theme in responses by research software funders. Software sustainability is a complex and, in some ways, an irresolvable challenge due to ever-shifting contexts on the broader research ecosystem (e.g., Howison, 2020; Katz, 2024). However, there is clearly a need for funders to address not only the technical aspects of software sustainability but also the social and professional infrastructure that bolsters long-term sustainability (e.g., Carver et al., 2021).

The theme of *Open Science* in the survey responses emphasized promoting open sharing, reuse, and accessibility of research software. This is achieved through strategic initiatives, including open science principles, explicit policy integration, support for diverse participants, and promoting open source practices. For example, funding open-source tools has been a major focus in the Chan Zuckerberg Initiative’s Essential Open Source Software for Science (EOSS) program, which has focused on software for biomedical research. A recent report on the EOSS program highlighted wide-ranging benefits from this investment in terms of scientific progress and diversifying scientific open source software teams and communities ( Hertweck et al., 2024). This suggests good alignment between funder priorities and the evidence base about what is yielding practical scientific and broader impacts.

Different practical ways of ensuring that research software contributes to such a healthy and open ecosystem are being discussed in the research software field (e.g., McKiernan et al., 2023; Sonabend et al., 2024; Sellanga et al., 2024). For example, applying FAIR (Findable, Accessible, Interoperable and Reusable) principles to research software has been highlighted as an essential step in the literature ( Barker et al., 2022). While our respondents mentioned concepts such as ‘accessibility’ and ‘reuse’, the term FAIR was not explicitly used. Overall, the survey responses evince a commitment to fostering an open, inclusive, and transparent research ecosystem. However, it is worth acknowledging that the focus on open source may be exaggerated in our purposive sample of funders already active in the research software arena, which is strongly oriented towards open source.

The theme of *building community and collaboration* was defined by efforts to enhance community engagement, foster international partnerships, and disseminate best practices. *Advancing research software funding* was also highlighted as a theme, with priorities including backing new software, strategic financial planning, and fostering collaborations. In these responses, funders emphasized the need for resources, strategic guidance, and joint efforts to enhance software impact. *Promoting the visibility and use of existing software* also emerged as a priority for research software funders. This included an interest in bolstering the awareness of available software by making it more prominent, cataloging and profiling available software resources, and encouraging software adoption. It has previously been noted that research software visibility can be bolstered through institutions that help to build community and collaboration, such as software sustainability institutes ( Katz et al., 2021).

A notable gap in the priorities identified by research software funders is the thorny challenge of bolstering recognition of research software contributions in academic hiring, promotion, and tenure ( Jensen & Katz, 2023a). This may appear to be outside the purview of research software funders, but such ‘people’ aspects are a clear priority for those who produce and maintain research software (e.g., Cohen et al., 2021; Lamprecht et al., 2022). A recent article summarized ‘ten simple rules’ for addressing this challenge, including ensuring that software contributions are recognized as key scholarly contributions, consistent with open science principles ( Puebla et al., 2024). While all of these rules/recommendations focus on the level of research institutions ( Barker et al., 2024), research funders can play a pivotal role in nudging the priorities and actions of such institutions. Therefore, research software funders should consider how they could help encourage research institutions to adopt a software-inclusive stance within hiring, promotion and tenure.

Much less frequently mentioned themes were *innovation* in research software, stressing the need to stay ahead of rapidly shifting trends in computing and the need to ensure that research software is secure from threats. Adapting research software good practices to advancements in AI and related developments is a need that has also been highlighted in the literature (e.g., Duarte et al., 2023), and it is an ongoing challenge facing the research software field. The theme of *security* in research software development and use was defined by a strong focus on ensuring data security, enhancing software security, and promoting security awareness training.

## Conclusion

The insights from research software funders reveal a commitment to enhancing the scientific landscape through targeted investments in software sustainability, open science, and community collaboration. Yet, the relative underemphasis on non-technical skills and social infrastructure suggests a need for a more holistic approach. To fully realize the potential of research software, funders must broaden their focus, integrating social and technical priorities to foster a resilient and dynamic research environment. By addressing such gaps, funders can help ensure that research software plays a more efficient, inclusive, sustainable, and impactful role within the scientific ecosystem.

## Ethics and consent

The University of Illinois Urbana-Champaign Institutional Review Board reviewed this research and determined it to be exempt (no. 24374) on September 11, 2023. All participants provided explicit informed written consent to participate in this study at the start of the online form used to gather the survey data. This included consent for data to be used for research purposes and to be published as open data after deidentification.

## Data Availability

Repository name: Zenodo Title of project: Strategic priorities and challenges in research software funding: Results from an international survey [Dataset]. DOI: https://doi.org/10.5281/zenodo.13852004 ( Jensen, 2024) This project contains the following underlying data:
• Survey data file: Anonymized responses from the international survey on strategic priorities and challenges in research software funding gathered from representatives of research funding organizations. Details of license: The anonymized survey data are available under the Creative Commons Attribution Non-Commercial No Derivatives 4.0 International license. Repository name: Zenodo Title of project: Strategic priorities and challenges in research software funding: Results from an international survey. DOI: https://doi.org/10.5281/zenodo.13852004 ( Jensen, 2024) This project contains the following extended data:
• Consent block from survey: The information and questions used to gather informed consent through the online survey have been uploaded as a document file. •
SURVEY DESIGN_Research_software_funder_survey_UIUC (upload to Zenodo).pdf Details of license: The anonymized survey data are available under the Creative Commons Attribution Non-Commercial No Derivatives 4.0 International license.
