# Supplementary material for: Strategic priorities and challenges in research software funding: Results from an international survey
Source: F1000Res. 2025 Jan 6;13:1447. Originally published 2024 Nov 29. [Version 2] doi: 10.12688/f1000research.155879.2 (PMC11799755; doi:10.12688/f1000research.155879.2)
Supplement: Peer review history

# Reviewer report: Reviewer response for version 2

**DOI:** 10.5256/f1000research.176306.r358849

**Published:** 2025-02-06

**Authors:** Rena Bakhshi

**Affiliations:** 1 Netherlands eScience Center, Amsterdam, The Netherlands

The current paper presents the results of a survey among research funders focusing on research software. In particular, the focus of the paper is around funders responses to the question "What are your organization’s top priorities related to research software?” The authors employ a qualitative research methodology for their analysis, and extract topics/themes from the survey results.

 While the topic and this empirical study are interesting, the paper is somewhat underwhelming as a "research article". Beyond reporting of the dataset (survey results), it is unclear to me how this paper is addressing the research question posed in the introduction.  The paper simply reports on the opinions of the respondents. What is missing is a deeper analysis, for instance, how these opinions relate to the policies of these organizations. For example, the authors write that "in sum, the responses signaled funders’ full-throated commitment to ensuring research software’s long-term viability and continuous improvement " but do not elaborate on relevant programs of those funders.

 The results section can be summarized in the form of a table to help readability.

Is the work clearly and accurately presented and does it cite the current literature?

Yes

If applicable, is the statistical analysis and its interpretation appropriate?

Not applicable

Are all the source data underlying the results available to ensure full reproducibility?

Partly

Is the study design appropriate and is the work technically sound?

Yes

Are the conclusions drawn adequately supported by the results?

Yes

Are sufficient details of methods and analysis provided to allow replication by others?

Partly

Reviewer Expertise:

research software engineering, project management, program management, computer science (formal methods)

I confirm that I have read this submission and believe that I have an appropriate level of expertise to confirm that it is of an acceptable scientific standard, however I have significant reservations, as outlined above.

# Reviewer report: Reviewer response for version 2

**DOI:** 10.5256/f1000research.176306.r356589

**Published:** 2025-02-05

**Authors:** Fabio Kon

**Affiliations:** 1 University of São Paulo, São Paulo, Brazil

This is an interesting paper showing the priorities of research funders concerning funding research software. The method is sound, and the anonymized data is presented in a public repository. The contribution is small but relevant and can help both funders engage in RS funding. It can also guide researchers when seeking funding to develop RS.

 The set of survey respondents is biased because it started with a list from the Research Software Alliance, but this is clearly stated in the paper.

 Thus, I believe the paper should be indexed in F1000research, but I suggest the following changes before indexing. 

 In the abstract, Results paragraph, the view is overly optimistic. Most funders probably don't value all these things; you got those skewed results because you used the ReSA list of contacts, as you correctly explain in Methods-Sampling. Somehow, this explanation should be reflected in the abstract, too.

 In the Methods-Survey design, it would be important to emphasize graphically the question "What are your organization.... software?"; e.g., by putting it in a box.

 In the Results section, you wrote that verbatim data extract in the form of a block quotation is shown. But, in fact, the reader can't see any block quotation, instead, a red text is displayed. I guess you might be referring to the latex command quotation that's obviously not seen by the reader. Probably, a \fbox command would work better.

 In the Results section, it would be interesting to know how often the topic was mentioned and/or how many respondents mentioned it.

 "the responses signaled funders' full-throated commitment to...". In fact, this doesn't show they're committed. It shows that they say this is something they look for in projects to be funded.

 In the discussion, I suggest revising the paragraph about Open Science as there's little connection between the beginning of the paragraph ("the theme of open science") and the example concerning EOSS. Funding OSS is a small part of Open Science in general, which that paragraph does not capture well.

Is the work clearly and accurately presented and does it cite the current literature?

Yes

If applicable, is the statistical analysis and its interpretation appropriate?

Yes

Are all the source data underlying the results available to ensure full reproducibility?

Yes

Is the study design appropriate and is the work technically sound?

Yes

Are the conclusions drawn adequately supported by the results?

Yes

Are sufficient details of methods and analysis provided to allow replication by others?

Yes

Reviewer Expertise:

Software Engineering, Data Science, Distributed Systems

I confirm that I have read this submission and believe that I have an appropriate level of expertise to confirm that it is of an acceptable scientific standard.

# Reviewer report: Reviewer response for version 2

**DOI:** 10.5256/f1000research.176306.r358851

**Published:** 2025-01-25

**Authors:** Claire Jean-Quartier

**Affiliations:** 1 Research Data Management, Graz University of Technology, Graz, Austria

Authors present qualitative survey results on research software funding focusing on related priorities by the funders. Research software has and is often neglected in publications, and one would guess that funders do not focus on or require researchers to plan for software produced and used during research projects to be sustainable. Still, this key aspect remains an open question and is addressed by the herein presented results from a qualitative survey.

 Though, it would be highly interesting to read about all results from the survey, it makes sense to split the more extensive survey and make use of the subsection regarding funders’ priorities in regard to research software, as has been done by the authors.

 The presented manuscript could be improved in terms of scientific completeness and comprehensibility, therefore suggesting the following major points:

 (1)  The manuscript seems to lack content in regard to the scientific background. It is recommended to extend the introduction section towards a more wholistic setting describing and reviewing software sustainability factors in general on the one side and general funders' priorities on the other side.

 (2) The results section could be made more comprehensible, since the so far used structural layout of text only, divided by numerous short paragraphs, does hinder readability. Results could be summarized in a table presenting key aspects, or something similar could be introduced to increase structural clarity and readability!

Is the work clearly and accurately presented and does it cite the current literature?

Partly

If applicable, is the statistical analysis and its interpretation appropriate?

Not applicable

Are all the source data underlying the results available to ensure full reproducibility?

Partly

Is the study design appropriate and is the work technically sound?

Yes

Are the conclusions drawn adequately supported by the results?

Yes

Are sufficient details of methods and analysis provided to allow replication by others?

Yes

Reviewer Expertise:

research data management, open science, bioinformatics, cancer science

I confirm that I have read this submission and believe that I have an appropriate level of expertise to confirm that it is of an acceptable scientific standard, however I have significant reservations, as outlined above.

# Reviewer report: Reviewer response for version 2

**DOI:** 10.5256/f1000research.176306.r356373

**Published:** 2025-01-22

**Authors:** Mario Coccia

**Affiliations:** 1 DSU, National Research Council Department of Engineering ICT and Technology for Energy and Transport, Rome, Lazio, Italy

Authors have partially clarified my questions presented in my first report.

Is the work clearly and accurately presented and does it cite the current literature?

Partly

If applicable, is the statistical analysis and its interpretation appropriate?

Partly

Are all the source data underlying the results available to ensure full reproducibility?

Partly

Is the study design appropriate and is the work technically sound?

Partly

Are the conclusions drawn adequately supported by the results?

Partly

Are sufficient details of methods and analysis provided to allow replication by others?

Partly

Reviewer Expertise:

science development and policies

I confirm that I have read this submission and believe that I have an appropriate level of expertise to state that I do not consider it to be of an acceptable scientific standard, for reasons outlined above.

# Reviewer report: Reviewer response for version 2

**DOI:** 10.5256/f1000research.176306.r356372

**Published:** 2025-01-07

**Authors:** Adrian Barnett

**Affiliations:** 1 Queensland University of Technology, Brisbane, Queensland, Australia

No further comments.

Is the work clearly and accurately presented and does it cite the current literature?

Yes

If applicable, is the statistical analysis and its interpretation appropriate?

Not applicable

Are all the source data underlying the results available to ensure full reproducibility?

Yes

Is the study design appropriate and is the work technically sound?

Yes

Are the conclusions drawn adequately supported by the results?

Partly

Are sufficient details of methods and analysis provided to allow replication by others?

No

Reviewer Expertise:

Statistics, research funding, meta-research

I confirm that I have read this submission and believe that I have an appropriate level of expertise to confirm that it is of an acceptable scientific standard.

# Reviewer report: Reviewer response for version 1

**DOI:** 10.5256/f1000research.171109.r344621

**Published:** 2024-12-13

**Authors:** Mario Coccia

**Affiliations:** 1 DSU, National Research Council Department of Engineering ICT and Technology for Energy and Transport, Rome, Lazio, Italy

**Strategic priorities and challenges in research software funding: Results from an international survey**

 The topics of this paper are  interesting, though well known. The structure and content must be revised, and results have to be  better explained by authors before to be reconsidered.

 Title has to be shorter and more specific of  the fields concerning funding research software. 

 Abstract has to clarify the goal, methods, empirical research and research policies to support funding of software. 

 Introduction has to better clarify the research questions of this study, considering that software is an intangible innovation, clarifying the sources that are problem driven in specific contexts, such as medicine, environmental sciences, etc. Role of metrics that can assess the effectiveness of research software as an open science, such as citations and their association with funding in the diffusion of science, etc. After that they can focus on the topics of this study to provide a correct analysis for fruitful discussion (See suggested readings that must be all read and used in the text). 

 Methods of this study is not clear. The section of Materials and methods must be re-structured with the following three sections:

 • **Sample and data.** The sample is not balanced between continents and also respondents and authors have to clarify how this is representative. 

 • **Measures of variables under study**

 • **Data analysis procedure**. 

 Insert a flow chart to specify the study design, with inclusion and exclusion criteria of organizations and respondents under study. 

 The identifications of themes and key aspects can be done with specific quantitative approaches, and not only qualitative, such also as entity linking, etc.... now the analysis is rather vague. 

**Results.**

 The sample can be represented with some figures to be clearer for readers. 

 Authors have to avoid subheadings that create fragmentation and confusion. If necessary, you can use bullet points (same comments for section of results and all sections).

 Results can be also systematized in some tables to be more effective in communicating results. 

 I suggest using different levels of description results from general to more specific themes, using tables to be more effective. 

**Discussion.** 

 First, authors have to synthesize the main results in a simple table to be clear for readers and then show what this study adds compared to other studies sone on these topics. 

 To reiterate, avoiding in the just mentioned sections, sub-headings that create fragmentation of the paper.

 The results should be better discussed considering the research fields because the sources of research software, their goal, their adopters change according to the research fields, such as biomedical research, environmental sciences, economic sciences, etc....role of research software, funding and citations for diffusion of open science should be useful to decide specific policies. A SWOT matrix with pros and cons can support managerial implications. 

 Conclusion has not to be a summary, but authors have to focus on manifold limitations of this study and based on results, provide best practices  of research policies to support the development of research software having practical implications for the diffusion of knowledge (also with citations) and development of science. 

 Overall, then, the paper is interesting, but theoretical framework is weak, and some results, too descriptive,  create confusion… structure of the paper has to be improved; study design, discussion and presentation of results have to be clarified using suggested comments.

 I strongly suggest improving the paper by using all comments (suggested papers included reading and using all) and maybe it can be considered. 

 Suggested readings.

 Afiaz, A., Ivanov, A.A., Chamberlin, J., ... Leek, J.T., Wright, C. 2024 (Ref -1). Best practices to evaluate the impact of biomedical research software-metric collection beyond citations, Bioinformatics, 40(8), btae469    

 Sochat, V., May, N., Cosden, I., Martinez-Ortiz, C., Bartholomew, S.    2022 (Ref-2). The Research Software Encyclopedia: A Community Framework to Define Research Software.Journal of Open Research Software, 10(1)    

 Coccia M. 2017. Sources of technological innovation: Radical and incremental innovation problem-driven to support competitive advantage of firms. Technology Analysis & Strategic Management, vol. 29, n. 9, pp. 1048-1061, https://doi.org/10.1080/09537325.2016.1268682\

 Cosden, I.A. 2022 (Ref-3). ,The Princeton University Research Software Engineering Group Model: Operational and Organizational Approaches.Computing in Science and Engineering, 24(5), pp. 24–31    

 Coccia M., Roshani S. 2024 (Ref-4). General laws of funding for scientific citations: how citations change in funded and unfunded research between basic and applied sciences. Journal of Data and Information Science, 9(1), 1–18. https://doi.org/10.2478/jdis-2024-0005

 Gomez-Diaz, T., Recio, T. 2022 (Ref-5). Research Software vs. Research Data II: Protocols for Research Data dissemination and evaluation in the Open Science context.F1000Research, 11, 117    

 Roshani S., Bagheri R., Mosleh M., Coccia M. 2021 (Ref-6). What is the relationship between research funding and citation-based performance? A comparative analysis between critical disciplines. Scientometrics 126, 7859–7874. https://doi.org/10.1007/s11192-021-04077-9

Is the work clearly and accurately presented and does it cite the current literature?

Partly

If applicable, is the statistical analysis and its interpretation appropriate?

Partly

Are all the source data underlying the results available to ensure full reproducibility?

Partly

Is the study design appropriate and is the work technically sound?

Partly

Are the conclusions drawn adequately supported by the results?

Partly

Are sufficient details of methods and analysis provided to allow replication by others?

Partly

Reviewer Expertise:

science development and policies

I confirm that I have read this submission and believe that I have an appropriate level of expertise to confirm that it is of an acceptable scientific standard, however I have significant reservations, as outlined above.

## References

[1] : Best practices to evaluate the impact of biomedical research software-metric collection beyond citations. *Bioinformatics* .2024;40(8) : 10.1093/bioinformatics/btae469 10.1093/bioinformatics/btae469 39067017 PMC11297485

[2] : The Research Software Encyclopedia: A Community Framework to Define Research Software. *Journal of Open Research Software* .2022;10(1) : 10.5334/jors.359 10.5334/jors.359

[3] : The Princeton University Research Software Engineering Group Model: Operational and Organizational Approaches. *Computing in Science & Engineering* .2022;24(5) : 10.1109/MCSE.2023.3264113 24-31 10.1109/MCSE.2023.3264113

[4] : General laws of funding for scientific citations: how citations change in funded and unfunded research between basic and applied sciences. *Journal of Data and Information Science* .2024;9(4) : 10.2478/jdis-2024-0005 71-89 10.2478/jdis-2024-0005

[5] : Research Software vs. Research Data II: Protocols for Research Data dissemination and evaluation in the Open Science context. *F1000Res* .2022;11: 10.12688/f1000research.78459.2 117 10.12688/f1000research.78459.2 36483317 PMC9706143

[6] : What is the relationship between research funding and citation-based performance? A comparative analysis between critical disciplines. *Scientometrics* .2021;126(9) : 10.1007/s11192-021-04077-9 7859-7874 10.1007/s11192-021-04077-9

# Response

**Published:** 2024-12-23

**Authors:** Eric Jensen

We are grateful for this report's helpful and constructive reviewer comments. The reviewer report requests some additional information and raises some points for discussion. While we don’t entirely agree with all points presented in this reviewer report, we have taken on board several points and made updates to the manuscript.

 Reviewer 2: “Title has to be shorter and more specific of  the fields concerning funding research software.”

 Response: The relevant research fields for the participating research funders was already presented in the ‘sampling subsection’ of the methods section: “Some participating funders cover a broad spectrum of disciplines, while others focus on a particular domain such as social science, health, environment, physical sciences or humanities.” Because this includes all research fields, it wouldn’t be appropriate to claim a narrower scope in the title.

 Reviewer 2: The reviewer makes several requests for a wider paper scope, e.g.: “Introduction has to better clarify the research questions of this study, considering that software is an intangible innovation, clarifying the sources that are problem driven in specific contexts, such as medicine, environmental sciences, etc. Role of metrics that can assess the effectiveness of research software as an open science, such as citations and their association with funding in the diffusion of science.”

 Response: These issues are beyond the scope of the current paper, which focuses on open-ended survey data from research funders. Imposing focus areas that did not emerge from these open-ended responses would be methodologically problematic. The research question currently stated in the manuscript is deliberately broad in line with the qualitative nature of the data that is being presented in the paper: “What are the top priorities of international research funding organizations related to research software?”

 Reviewer 2: “Methods of this study is not clear. The section of Materials and methods must be re-structured with the following three sections: […] Insert a flow chart to specify the study design, with inclusion and exclusion criteria of organizations and respondents under study. 

 The identifications of themes and key aspects can be done with specific quantitative approaches, and not only qualitative, such also as entity linking, etc.” 

 Response: The current Methods section structure follows the journal guidelines and is appropriate for a qualitative survey study like this. A purely qualitative analysis and reporting of results is permitted by this journal; adding quantitative methods is not required.

 Reviewer 2: “Authors have to avoid subheadings that create fragmentation and confusion. If necessary, you can use bullet points (same comments for section of results and all sections).

 Results can be also systematized in some tables to be more effective in communicating results. 

 I suggest using different levels of description results from general to more specific themes, using tables to be more effective.”

 Response: We appreciate the reviewers ideas about how to present the results. However, the current structure is in line with accepted practices in qualitative research. Each subheading represents a distinct theme, so it would be inappropriate to force them together.

 Reviewer 2: Raises concerns about the Discussion section structure

 Response: While the reviewer’s feedback about the Discussion section is noted, it is presented in a manner consistent with the journal guidelines and normal social science practices. Adding a table, for example, would be out of line with typical Discussion section approaches for this kind of paper.

 Suggested readings: Because none of the suggested readings directly relate the research software funding, these papers have not been added to the citation list despite the reviewer’s insistence on adding citations to the reviewer’s published work.

# Reviewer report: Reviewer response for version 1

**DOI:** 10.5256/f1000research.171109.r344615

**Published:** 2024-12-13

**Authors:** Adrian Barnett

**Affiliations:** 1 Queensland University of Technology, Brisbane, Queensland, Australia

The paper examines funding for software. Software must compete against other priorities, e.g., equipment. It also must compete against other more “exciting” ideas, and hence may be neglected despite its importance to research. As an example, here’s a relevant and recent skeet from the software goliath Hadley Wickham (27 November 2024):

 “I had zero luck with grants. People really didn’t get why what I did was important and never figured out how to explain it to them.” https://bsky.app/profile/hadleywickham.bsky.social/post/3lbumdqcmcc2r

 And an interesting reply to Hadley’s skeet: “lol thinking about you burying the tidyverse into a corner of aim 3 for an NIH grant on some very specific cardiovascular outcome, which is how most infrastructure work gets funded” https://bsky.app/profile/travisgerke.bsky.social/post/3lca7rkqv222z

 I don’t usually quote skeets or tweets in peer reviews, but these are good anecdotal examples of how software can miss out to other priorities. It also highlights how software is often funded as an add-on rather than the main idea.

 Without breaking confidentiality, some more summary details on the funders would be useful to contextualise their answers and assess the generalisability of the results. Are the funders predominantly in science, engineering, or health and medicine? Or do they cover all these areas. What’s the total funding awarded per year? How many scientists per year do they support?

 The sampling frame could be biased towards funders with a greater interest in software as: “participation in the Research Software Association (ReSA) and responsibility for research software funding known to the authors.” This should be discussed as a limitation. It would also be useful to describe who ReSA are.

 The questions asked the funders about priorities, but did not ask for actual financial support. Funders can have many priorities, but with strong competition for funding they might not all get funded. Money spent on software would be the best measure of support. I realise this can’t be collected now, but some discussion of this would be useful.

 The quotes from funders were generally very positive and I expected to see something in the results on the challenges of funding software. This prompted my previous question on wanting to know more about the funders, as I wondered if they were an atypical group of funders with a particular interest in software. Perhaps the funders were not prompted for their negative experiences?

 I was surprised that there was nothing on partnering with industry. In Australia the funding schemes that require partnerships with industry (or government) have a much higher success rate than standard schemes. Given the potential commercial benefits of software, partnerships would be an option for many software applications.

 Minor comments:

- Sampling section (and elsewhere): “87.8%” – no need for a decimal place, see: Cole T. Too many digits: the presentation of numerical data, DOI: 10.1136/archdischild-2014-307149
- The key question “What are your organization’s top priorities related to research software?” has no time frame. So I presume it was current priorities only? Although the question: “Has your organization established any policies, initiatives or programs aimed at supporting research software?” covers all times. A consistent time frame may have been better, for example, “in the last 5 years”.
- Discussion: I agree that funders have a part to play in increasing the recognition of software. Funders can be influential in how science is recognised and rewarded. The COARA initiative is likely relevant here, as it discusses needing to recognise the “diversity of contributions” which includes those scientists who create valuable software ( https://coara.eu/agreement/the-commitments/).

Is the work clearly and accurately presented and does it cite the current literature?

Yes

If applicable, is the statistical analysis and its interpretation appropriate?

Not applicable

Are all the source data underlying the results available to ensure full reproducibility?

Yes

Is the study design appropriate and is the work technically sound?

Yes

Are the conclusions drawn adequately supported by the results?

Partly

Are sufficient details of methods and analysis provided to allow replication by others?

No

Reviewer Expertise:

Statistics, research funding, meta-research

I confirm that I have read this submission and believe that I have an appropriate level of expertise to confirm that it is of an acceptable scientific standard, however I have significant reservations, as outlined above.

## References

[1] : Too many digits: the presentation of numerical data. *Arch Dis Child* .2015;100(7) : 10.1136/archdischild-2014-307149 608-9 10.1136/archdischild-2014-307149 25877157 PMC4483789

# Response

**Published:** 2024-12-23

**Authors:** Eric Jensen

We are grateful for this report's helpful and constructive reviewer comments. The reviewer report requests some additional information and raises some points for discussion. The details for how these points have been addressed in the new version of the manuscript are presented below:

 Reviewer 1: “Without breaking confidentiality, some more summary details on the funders would be useful to contextualise their answers and assess the generalisability of the results. [a] Are the funders predominantly in science, engineering, or health and medicine? Or do they cover all these areas. [b] What’s the total funding awarded per year? How many scientists per year do they support?”

 Response: [a] This information was already presented in the ‘sampling subsection’ of the methods section: “Some participating funders cover a broad spectrum of disciplines, while others focus on a particular domain such as social science, health, environment, physical sciences or humanities.” [b] this information was not collected in the survey.

 Reviewer 1: “The sampling frame could be biased towards funders with a greater interest in software as: “participation in the Research Software Association (ReSA) and responsibility for research software funding known to the authors.” This should be discussed as a limitation. It would also be useful to describe who ReSA are.”

 Response: This is already discussed as a limitation. However, more limitations content has now been added to the methods section: “ReSA is an international organization involving research policymakers, funders and organizations that recognize software’s importance in their work. This means that a ReSA-focused sample is likely to over-represent research funders who already view research software as important. For this reason, this paper does not claim that its findings generalize to the global population of research funders; rather, the claims focus on current patterns in research software funding.”

 Reviewer 1: “The questions asked the funders about priorities, but did not ask for actual financial support. […] Money spent on software would be the best measure of support. I realise this can’t be collected now, but some discussion of this would be useful.”

 Response: This point is now addressed in the survey design subsection of the Methods section: “Clearly, potentially relevant variables were not included in the survey design for this exploratory study. These include details on the exact amount of funding spent on research software and other details about which many research funders would be unable to readily report either because the information is not actively tracked or relevant data do not currently exist.”

 Reviewer 1: “I was surprised that there was nothing on partnering with industry. In Australia the funding schemes that require partnerships with industry (or government) have a much higher success rate than standard schemes. Given the potential commercial benefits of software, partnerships would be an option for many software applications.”

 Response: There is an important distinction between research software and software for more general use. Research software, per se, often attracts less industry interest than some other software domains due to its tendency to have a narrower market/commercial potential.
